# Under the mask: The double-edged sword effect of leader self-sacrifice on employee work outcomes

**DOI:** 10.3389/fpsyg.2023.1052623

**Published:** 2023-02-06

**Authors:** Yu-Chen Jiao, Yu-Chen Wang

**Affiliations:** ^1^Management School, Jinan University, Guangzhou, China; ^2^School of Business Administration, Lanzhou University of Finance and Economics, Lanzhou, China; ^3^School of Tourism, Hainan University, Haikou, China

**Keywords:** leader self-sacrifice, authenticity attribution, trust in leader, perceptions of leader hypocrisy, employee task performance, organizational citizenship behavior

## Abstract

Building on attribution theory, this study applied regression analysis and explored the double-edged sword effect of leader self-sacrifice behavior on employee work outcomes, thus revealing the potential negative impacts of such behavior. Specifically, when leadership self-sacrifice was met with low employee authenticity attribution, we found that employees tended to perceive leadership as hypocritical, thus reducing their organizational citizenship behavior. By contrast, when leaders’ self-sacrifice behavior was met with high employee authenticity attribution, employees tended to trust the leader and improve their task performance. Given these findings, we challenge the general scholarly consensus on leadership self-sacrifice behavior, enrich the current literature on leadership self-sacrifice, and emphasize the important role of employee attribution in the relevant leadership process.

## 1. Introduction

The corridors of history are filled with examples of leaders, and the legacy left by many outstanding leaders is that they sacrificed themselves for the interests of the group (Yang F. et al., 2022). We have seen many entrepreneurs inspire followers to join them in achieving organizational goals by inspiring them at the expense of their own interests. To depict and summarize this self-sacrificing behavior of leadership, Choi and Mai-Dalton (1998) defined it as a leadership trait that voluntarily delays or waives personal interests or privileges for the realization of organizational goals and collective well-being. Over the last two decades, scholars have extensively focused on the subject of leader self-sacrifice. Numerous researches have shown that leaders’ self-sacrifice behavior arouses positive emotions in employees (De Cremer et al., 2009; Vianello et al., 2010), which improves helping behavior (Liu et al., 2022), work engagement (Mostafa and Bottomley, 2020) and task performance (Jiang et al., 2019). In short, there seems to be a common consensus among scholars that employees can be encouraged by leaders’ self-sacrifice behavior to produce better work results.

Although the above studies have confirmed that employees can have a positive response to a leader’s self-sacrifice behavior—that is, they can demonstrate good work results—these studies also reveal that scholars know little about the negative impact it may cause (Liu et al., 2022). The present study argues that scholars pay too much attention to the positive impact of leadership self-sacrifice behavior while ignoring its possible negative impact and its potential double-edged sword effect. We believe that the following two reasons have induced to this situation. First, scholars have neglected to consider that self-sacrifice behavior may be a political manipulation of leadership (Greenbaum et al., 2015), that is, leaders’ self-sacrifice does not necessarily arise from their real or authentic self. In fact, self-interest is at the core of many human behaviors, but self-sacrificing behavior by leaders contradicts this principle, which will induce employees to doubt the leader’s motive (Carlson et al., 2022). Then, they generally use means of attribution to explore the motives behind the leadership behavior. This brings us to find the second research gap, which is that scholars have ignored the importance of employee attribution of leadership behavior. Previous research has largely been based on the hypothesis of leadership-centric perspective (Xu et al., 2022; Yang F. et al., 2022). In other words, scholars believe that as long as leaders demonstrate self-sacrifice behaviors, employees will have a positive psychological state, thus actively improving their work results. However, this assumption is one-sided and incomplete. Because attribution theory holds that individuals have a desire to explore the motivations of others’ behavior (Ferris et al., 1995). Therefore, employees will attribute leadership behavior in the process of interaction with the leaders, and the attribution results will impact their subsequent psychological state and behavior (Dasborough and Ashkanasy, 2002). Based on the above two gaps, we observed that gaps in the previous research left a possibility for us to explore the negative impact of leadership self-sacrifice behavior and its double-edged sword effect. That is, exploring the double-edged sword effect of leadership self-sacrifice behavior on employees’ work results from the perspective of employees’ authenticity attributions of this self-sacrifice behavior can both make up for the lack of research on the dark side of leadership self-sacrifice behavior and help us understand this behavior from a balanced and dialectical perspective.

Employees’ attributions of the authenticity of leaders’ self-sacrifice behavior involve employees judging the authenticity of the actual motivation behind this behavior (Biermeier-Hanson et al., 2020; Long, 2021). Attribution theory posits that all humans are born psychologists; that is, they are born with the natural motivation to find and explain the causes of related events (Heider, 1958). In fact, as abovementioned, self-sacrifice behavior is a leadership behavior that is largely contrary to individual intuition. Therefore, when leaders demonstrate sacrifice behaviors, it can easily cause employees to question the authenticity of these leaders’ motivation (Choi and Mai-Dalton, 1998). When employees believe that this behavior is of low authenticity, they will think that the self-sacrifice behavior of the leaders are tactical behaviors or a type of hypocrisy that is inspired by the external environment (Cha and Edmondson, 2006). As such, employees may believe that leadership is hypocritical. When employees perceive leadership as hypocritical, they experience negative emotions and a sense of uncertainty, and may even think that they are being used by leaders. Consequently, they may engage in retaliatory slightly behavior, that is, employees may reduce their organizational citizenship behavior (Bharanitharan et al., 2021). When employees attribute leaders’ self-sacrifice behavior to, authenticity they find that this behavior originates from the leader’s inherent personality traits (Martinko and Gardner, 1987) and is inspired by their personal charm. Trust in leaders is, thus, stimulated (Jung and Avolio, 2000). Out of the principles of reciprocity, as employees’ trust in leaders improves, they will increase their willingness to return these leaders; therefore, they will take the initiative to improve their task performance level because achieving organizational performance goals is what leaders pursue. In sum, our study holds that a leader’s self-sacrifice behavior has variable impacts on employee work outcomes depending on whether the employees believe the leader’s behavior is authentic. This is because employees have different attributions for the authenticity of leadership self-sacrifice behavior, leading either to the psychological state of trust in the leader or an perceptions of leadership hypocrisy, which result in different work outcomes.

Working outside traditional leadership-centric perspective, this study adopted attribution theory to take the perspective of employees’ attributions of the authenticity of leadership self-sacrifice behavior. In this regard, we balanced and dialectically responded to the controversy on altruistic and egoistic motives behind leadership behavior through an investigation of the double-edged sword effect of leadership self-sacrifice behavior, thus challenging the current scholarly consensus. At the same time, we identified the mediating roles of trust in leaders and leadership hypocrisy, with further clarification of the mechanism in the relationship between leadership self-sacrifice behavior and employee work results. In sum, this study enriches the application of attribution theory in organizational behavior research, broadens the research perspective on leadership self-sacrifice behavior, and provides inspiration and reference for leaders who aim to use their own self-sacrifice behaviors to better motivate employees. The subsections below discuss our theoretical framework and list the research hypotheses.

### 1.1. Theoretical framework

According to attribution theory, leaders attribute and interpret the motivations behind employee behavior (Martinko et al., 2007, 2011). The results of such attributions lead to different psychological states in the leader, thus inducing different responses to employees’ behaviors. In particular, when employees’ behaviors are unexpected or contrary to individual intuition, it is more likely to cause leaders to attribute a judgment of authenticity to these behaviors (Weiner, 1985). For example, when employees attempt to ingratiate themselves with leaders, leaders may doubt the authenticity of their behavior, thus attributing employee behavior (Turnley et al., 2013; Bolino et al., 2016). In the leadership research, scholars have paid significant attention to leaders’ attributions of employees’ behavior; however, less attention has been paid to employees’ attributions of leadership behavior. In fact, the relationship between leaders and subordinates is essentially a binary interactive relationship (Dasborough and Ashkanasy, 2002) which means that employees are both recipients and observers of leadership behavior, they also attribute leadership behavior (Liu et al., 2012; Qin et al., 2020; Zhao et al., 2022). The effectiveness of leadership behavior is usually perceived by employees, meaning that the effectiveness of leadership self-sacrifice behavior depends largely on employees’ perceptions and attributions of the authenticity of this behavior. Greenbaum et al. (2015) proposed that a leader’s self-sacrifice is likely to be a means of manipulating employees for their own interests. This behavior is not necessarily authentic, which makes it possible for employees to attribute a leader’s self-sacrifice behavior from the perspective of authenticity. Based on this, this study focused on the interaction between leaders’ self-sacrifice behavior and employees’ attribution of the authenticity of leadership behavior and its effect on employees’ psychological states and work results.

### 1.2. Research hypotheses

#### 1.2.1. The interactive effects of leader self-sacrifice and employee authenticity attribution on leader hypocrisy

Perceptions of leader hypocrisy mean that employees believe that a leader’s behavior is inconsistent with the values they espouse (Cha and Edmondson, 2006; Bharanitharan et al., 2021). In fact, leaders hope that through displays of self-sacrificial behavior, their subordinates will perceive their charm and that they will convey the values of self-sacrifice (De Cremer and Van Knippenberg, 2004; Choi and Yoon, 2005). However, according to attribution theory, whether the personal charm shown by leaders and the values they convey will be perceived and accepted by employees depends on employees’ understanding and attributions of leadership behavior (Xing et al., 2021). Only when the leader is a true example can his values be recognized by his followers and reflected in their behavior (Saeed et al., 2022). Based on this logic, this study posits that whether leaders’ self-sacrifice behavior can motivate employees depends on employees’ attribution of authenticity to such leadership behavior. According to House (1992), leaders may have selfish intentions despite their demonstrations of self-sacrifice. In similar research, Choi and Mai-Dalton (1998) reported that leaders may exhibit self-sacrifice as part of their impression management strategies and/or given the desire to manipulate employees. Thus, leadership self-sacrifice may be driven by motivations for self-interest, which may influence employees to develop suspicions about the motivation for such behavior, and thus make attributions of inauthenticity. When employees believe that the leader’s behavior is of low authenticity, they will perceive the behavior as an effort for personal gain or employee manipulation. In such cases, self-sacrifice behaviors are inconsistent with the leader’s own values, meaning that employees will gauge the leader as hypocritical. However, when employees believe that leadership behavior is of high authenticity, they will perceive this behavior as consistent with the values the leader embraces and conveys (Greenbaum et al., 2015) and, therefore, not judge the leadership as hypocritical. Based on this, we propose the following hypothesis:

*H1*: The interaction effect between self-sacrificial leadership and employee authenticity attribution positively impacts employee perceptions of leadership hypocrisy. With lower authenticity attributions, employees will believe that the leadership is more hypocritical; conversely, with higher authenticity attributions, employees will believe that the leadership is less hypocritical.

#### 1.2.2. Leader hypocrisy and employee organizational citizenship behavior

According to the above derivation, leadership hypocrisy is a kind of perception generated in employees after they make attributions of leadership self-sacrifice behavior (Bharanitharan et al., 2021). According to the logic of attribution theory, employees will decide on their subsequent behavior and response according to their own attributions and the resulting psychological state. In this context, employees who perceive leadership hypocrisy experience a sense of awakening (Cha and Edmondson, 2006). This is characterized by negative emotions such as disappointment, frustration, anger, and even feelings of betrayal. To deal with these negative emotions, employees may retaliate (Bharanitharan et al., 2021; Zhao et al., 2022); however, they do not want this retaliation to cause serious harm to their careers (Bruk-Lee and Spector, 2006), so they may actively reduce their extra-role behavior, that is, organizational citizenship behavior. Second, when employees perceive that a leader’s true intentions are inconsistent with the values they hold, they perceive their leader as a “hypocrite,” which can make them uncomfortable (Simons, 2002; Greenbaum et al., 2015; Zhao et al., 2022) and reduce their liking of the leader (Mharapara et al., 2022). They will then reduce their organizational citizenship behavior because work output that exceeds the prescribed range may become a tool for leaders to seek personal gain for themselves, a scenario that employees do not want (Williams and Anderson, 1991). Finally, leadership hypocrisy undermines employees’ quest for stability (Mharapara et al., 2022). Since a leader’s true intentions are elusive and may change at any time, it becomes difficult for a good relationship to be established between employees and leaders. Studies have confirmed that only in a high-quality leader-member relationship will employees actively engage in extra-role behavior (Ilies et al., 2007; Wang et al., 2017). In sum, employees who perceive leader hypocrisy may not exhibit organizational citizenship behavior. Based on this evidence, we propose the following hypothesis:

*H2*: The perception of leader hypocrisy mediates the interaction effect between leader self-sacrifice and employee authenticity attribution on organizational citizenship behavior. When employee authenticity attribution is low, the indirect effect is negative; when employee authenticity attribution is high, the indirect effect does not exist.

#### 1.2.3. The interactive effects of leader self-sacrifice and employee authenticity attribution on trust in leaders

In the field of organizational behavior, trust is considered an important factor for maintaining organizational effectiveness and promoting organizational survival. In particular, subordinate trust in leader is one of the most important mediating variables that connect the leadership process with organizational performance (Zada S. et al., 2022). For leaders, self-sacrifice is only accepted and recognized by subordinates who trust them. As such, this study investigated whether leader self-sacrifice was trusted by employees.

We suggest that when employees attribute a leader’s self-sacrifice is highly authentic, it will stimulate their trust in the leader. From the perspective of leadership trait theory, employees’ trust in leadership can be conceptualized as faith and loyalty to leadership (Podsakoff et al., 1990). When employees attribute leaders’ self-sacrifice behavior as real, it means they believe that the leaders’ self-sacrifice originates from their altruistic tendencies (Matteson and Irving, 2006). An altruistic character and altruistic motivation are both virtues and valuable traits. Some philosophers even believe that self-sacrifice arises from altruistic motivation forms the “love” of any relationship, including the “Neighbor-love” (Dalferth, 2010). We posit that on this basis, employees can perceive a leader’s personality and charm through their self-sacrifice (Choi and Mai-Dalton, 1998), which will generate belief in the leader. At the same time, leaders who show self-sacrifice behavior at work usually undertake more challenging work and tasks than employees and give up personal interests for the sake of organizational or collective development (De Cremer and Van Knippenberg, 2004). Therefore, when employees attribute leadership behavior as highly authentic, they will believe that the leader’s behavior aims to achieve the development of the team and the collective, thus, believing that they can also develop and benefit from this behavior as part of the team (Li et al., 2014). This substantial benefit will encourage employees to remain loyal to their leadership. In addition, some scholars have found through semi-structured interviews that the higher the employee’s perception of the authenticity of their leader, the more likely they are to trust this leader (Soderberg and Romney, 2022). To sum up, when employees believe that their leader’s self-sacrificial behavior is highly authentic, it inspires greater trust in the leader. By contrast, employees believe that leadership self-sacrifice is merely a strategic tool in cases of lower perceived authenticity, which reduces their level of trust (Howell and Avolio, 1992; Sparrowe, 2005). Based on this evidence, we propose the following hypothesis:

*H3*: The interaction effect between leadership self-sacrifice behavior and employee authenticity attribution positively affects trust in leaders. With higher authenticity attributions, employees have higher trust in the leader; conversely, with lower authenticity attributions, employees have lower trust in the leader.

#### 1.2.4. Trust in leader and employee task performance

Trust is a psychological state in which individuals are willing to expose their weaknesses to others; they are not worried about being exploited due to positive expectations that are based on the intentions and behaviors of others (Rousseau et al., 1998). When employees believe that a leader is trustworthy, they believe that the leader’s abilities and personality are also trustworthy (Kirkpatrick and Locke, 1996; Podsakoff et al., 1990). This reduces employees’ uncertainty about leaders’ behavior and, in turn, increases their willingness to accept the influence leaders have on them (Dirks and Ferrin, 2002), thereby allowing them to recognize and work toward the organizational goals set by leaders (Dirks and Ferrin, 2002; Um-e-Rubbab et al., 2021). Thus, when employees believe that a leader’s self-sacrifice behavior is authentic and trust the leader, they will accept the collective goals to which the leader gives importance (Fatima et al., 2017), which thus, actively improves their task performance level. Because organizational goals depend on the performance realization of individual goals. In addition, one of the important reasons for leaders to demonstrate self-sacrificial behavior is that employees can work with them to achieve organizational interests and organizational goals. When employees trust leaders, they will likely want to return the leader’s sacrifice based on the principle of reciprocity in social exchange theory (He et al., 2022; Zada M. et al., 2022); therefore, employees will take the initiative to improve their task performance level and achieve organizational goals as a kind of reward for their leader (Yang B. et al., 2022). By contrast, when employees do not attribute their leader’s self-sacrifice behaviors as authentic, their trust in the leader is reduced, and they will focus more on “covering their back.” At the same time, they will search for inauthentic behaviors or information from leaders at work, thereby reinforcing their previous interpretations and attributions of leadership behavior. Meanwhile, these searching behaviors undoubtedly consume energy and reduce employee task performance (Choi and Mai-Dalton, 1998; Mayer and Davis, 1999). Based on this evidence, we propose the following hypothesis:

*H4*: Trust in leaders mediates the interaction effect between leader self-sacrifice and employee authenticity attribution on employee task performance. With high authenticity attributions, the mediating effect is high; with low authenticity attributions, the mediating effect does not exist.

**Figure 1:**
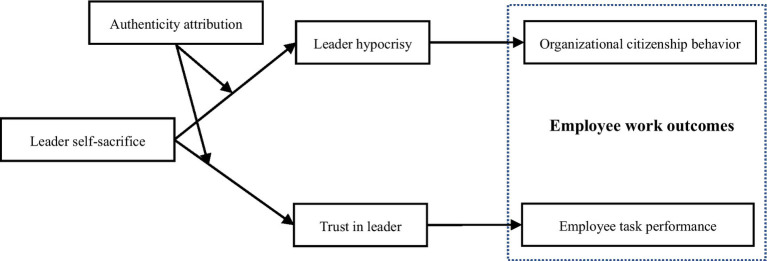
Illustrates this study’s theoretical model.

The theoretical model of this study is shown in Figure 1.

## 2. Materials and methods

### 2.1. Sample selection and data collection

We collected data by conducting a questionnaire survey among six enterprises in Shaanxi and Jiangxi, China. These enterprises were positioned in different fields, including the construction industry, electronic processing industry, and scientific research. The survey was conducted in an offline format, with questionnaires collected at two time points using a leader–member matching method. At time node T1, we conducted a grassroots questionnaire among employees at the above enterprises, with items on personal information, leadership self-sacrifice, and authenticity attribution. These were collected on the spot. At time node T2 (1 month after T1), we distributed another round of questionnaires to the same employees, with items on trust in leaders, leadership hypocrisy, and organizational citizenship behavior. A second questionnaire including the employee’s name was issued to the corresponding supervisor of the employee, who completed the questionnaire, with items on the leader’s personal information and employee task performance; at this time, the leader evaluated the employee’s task performance and returned this information on the spot. On average, about 83 employee questionnaires were distributed for each enterprise, and about 14 leadership questionnaires for each enterprise. We distributed questionnaires to a total of 500 employees and 85 mid-level leaders, with each leader, therefore, corresponding to an average of five to six employees. On average, each enterprise received about 75 employee questionnaires and about 13 questionnaires from leaders. Ultimately, we collected 451 employee questionnaires (recovery rate of 90.20%) and 79 leader questionnaires (recovery rate of 92.94%). After excluding responses that could not be matched and had obvious problems with consistency and other selection issues, we analyzed data from 412 valid employee questionnaires (effective rate of 91.35%) and 75 valid leader questionnaires (effective rate of 94.94%).

Descriptive statistics showed that the sample of 412 employees contained 207 (50.20%) men and 205 (49.80%) women. As for age, 99 (24.03%) were under 25 years, 76 (18.45%) were between 26 and 30 years, 73 (17.72%) were between 31 and 35 years, 86 (20.87%) were between 36 and 40 years, and 78 (18.93%) were over 40 years. In terms of their qualifications, 102 (24.75%) had junior high school qualifications or below, 72 (17.48%) had high school qualifications, 72 (17.48%) had college qualifications, 77 (18.69%) had undergraduate degrees, and 89 (21.60%) had master’s degrees or above. As for working time between employees and their current supervisors, 81 (19.66%) reported less than 1 year, 78 (18.93%) reported 1 to 2 years, 85 (20.63%) reported 2 to 3 years, 76 (18.45%) reported 3 to 4 years, and 92 (22.33%) reported more than 4 years. Of the 75 respondents in the leader sample, 68 (90.67%) were men and seven (9.33%) were women, with an average age of 45.30 years; 59 (78.67%) had bachelor’s degrees or above.

### 2.2. Variable measurements

The measurement tools in this study included mature scales developed outside of China. The “translation-back translation” procedure was used to translate English scales into Chinese versions (Brislin, 1970). We invited two professors of linguistics to assist us. Items were answered using a 5-point Likert scale ranging from 1 (very inconsistent) to 5 (very consistent).

The employees assessed leader self-sacrifice on the five-item scale from De Cremer and Van Knippenberg (2004) (e.g., “When necessary, my leader is willing to sacrifice their own interests to safeguard the interests of employees”). In this study, the scale had a Cronbach’s ɑ of 0.916.

The leaders assessed employee task performance on the four-item work effort scale developed by Chen et al. (2002) (e.g., “The employee always completes the task on time”). In this study, the scale had a Cronbach’s ɑ of 0.90.

Trust in leaders was measured using the six-item scale from Podsakoff et al. (1990) (e.g., “I fully believe that they are a person of integrity and honesty”). In this study, the scale had a Cronbach’s ɑ of 0.92.

Perception of leader hypocrisy was measured using the four-item scale developed and compiled by Greenbaum et al. (2015) (e.g., “My leader often asks me to follow the rules, but they cannot do it themselves”). In this study, the scale had a Cronbach’s ɑ of 0.90.

Employee authenticity attribution was measured with reference to the scale from Long (2021). The following prompt was included: “To what extent does your leader engage in each of the following behaviors because it is who they are/because it is in their nature to act that way.” This was followed by the five-item Leadership Self-Sacrifice Scale used by De Cremer and Van Knippenberg (2004). In this study, the scale had a Cronbach’s ɑ of 0.93.

Organizational citizenship behavior was measured using the nine-item scale from Farh et al. (2004) (e.g., “Even if no attention or no evidence is reliable, I will always abide by the company’s regulations”). In this study, the scale had a Cronbach’s ɑ of 0.90.

Since demographic characteristics may impact measurements, we asked employees for their gender and age; following previous research, we used these as control variables to exclude their impacts on responses to the leadership items (Eagly and Karau, 2002; Anderson et al., 2017). Studies have also shown that employees are more affected by leaders with whom they work longer hours (Maslyn and Uhl-Bien, 2001). As such, we used the number of years of the employees had worked with their leaders as a control variable. Finally, employee education levels impact their work results (Fox et al., 2012; Jacobson et al., 2015); thus, we controlled for this variable.

## 3. Results

### 3.1. Discriminant validity test and common method bias

Since all the study scales were mature, their content validity was verified. We used AMOS 24.0 to conduct a confirmatory factor analysis aimed at verifying discriminant validity between variables. As shown in Table 1, the six-factor model had the best fit (GFI = 0.84, AGFI = 0.81, IFI = 0.92, TLI = 0.92, CFI = 0.92, RMSEA = 0.06), indicating good discriminant validity for each variable.

**Table 1:** Confirmatory factor analysis results.

Model	χ^2^	df	χ^2^ /df	CFI	GFI	IFI	TLI	RMSEA	AGFI
Six-factor model	1180.92	449	2.63	0.92	0.84	0.92	0.92	0.06	0.81
Five-factor model	2093.28	454	4.61	0.83	0.73	0.83	0.81	0.09	0.68
Four-factor model	3115.49	458	6.80	0.72	0.61	0.72	0.70	0.12	0.52
Three-factor model	4351.38	461	9.44	0.59	0.51	0.59	0.56	0.14	0.43
Two-factor model	6330.78	463	13.67	0.38	0.42	0.38	0.37	0.18	0.36
Single-factor model	7950.12	464	17.13	0.20	0.34	0.12	0.16	0.20	0.25

Six-factor model, leadership self-sacrifice + trust in leader + leader hypocrisy + authenticity attribution + task performance + organizational citizenship behavior; Five-factor model, leadership self-sacrifice + leader hypocrisy, trust in leader, authenticity attribution, task performance, organizational citizenship behavior; Four-factor model, leadership self-sacrifice, trust in leader + leader hypocrisy + task performance, authenticity attribution, organizational citizenship behavior; Three-factor model, leadership self-sacrifice + trust in leader + leader hypocrisy, authenticity attribution, organizational citizenship behavior; Two-factor model, leadership self-sacrifice, trust in leader + leader hypocrisy + authenticity attribution + task performance + organizational citizenship behavior; Single-factor model, leadership self-sacrifice + trust in leader + leader hypocrisy + authenticity attribution + task performance + organizational citizenship behavior.

This study used self-reported data, which are prone to common method biases (Podsakoff et al., 2003). Therefore, we conducted the Harman single factor test, which showed that the first principal component obtained without rotation accounted for 19.70% of the total factor load; this did not exceed the critical value of 40%, indicating that the common method bias problem was not serious. After adding the common method factor, we then compared the original confirmatory model M1 and model M2 of the common method factor, as follows: ΔRMSEA = 0.01, ΔGFI = 0.01, ΔAGFI = 0.02, ΔCFI = 0.02. The change of each fitting index did not exceed 0.02, indicating that the model fitting degree did not improve significantly after adding the common method factor, which further indicated that the common method deviation problem involved in this study was not serious. This did not affect the statistical analysis results to a problematic level (Bagozzi and Yi, 1990).

### 3.2. Descriptive statistics and correlation analysis

Table 2 shows the correlation coefficients and descriptive statistics between variables. According to the preliminary analysis, leader self-sacrifice was significantly and positively correlated with both trust in the leader (*r* = 0.34, *p* < 0.001) and leader hypocrisy (*r* = 0.26, *p* < 0.001). We found a significant positive correlation between trust in the leader and employee task performance (*r* = 0.25, *p* < 0.001) and a significant negative correlation between leadership hypocrisy and organizational citizenship behavior (*r* = −0.22, *p* < 0.001). These results provided a basis for the next regression analysis.

**Table 2:** Variable descriptive statistics and related results analysis.

	1	2	3	4	5	6	7	8	9	10
1 Sex	–									
2 Age	0.08	–								
3 Education	0.04	−0.01	–							
4 Working time with supervisor	−0.07	0.03	0.03	–						
5 Leader self-sacrifice	−0.01	−0.06	−0.06	0.01	–					
6 Trust in leader	0.01	−0.02	−0.08	0.21	0.34^***^	–				
7 Perception of leadership hypocrisy	−0.01	0.02	−0.02	0.03	0.26^***^	0.01	–			
8 Authenticity attribution	−0.05	−0.01	0.07	0.00	0.30^***^	0.18^***^	0.14^**^	–		
9 Task performance	−0.01	−0.07	−0.05	−0.08	0.00	0.25^***^	−0.13^**^	0.05	–	
10 Organizational citizenship behavior	−0.04	−0.06	−0.05	−0.05	0.02	0.09	−0.22^***^	−0.01	0.15^**^	–
*M*	0.50	2.92	2.95	3.05	3.35	3.67	3.22	3.39	3.52	3.79
*SD*	0.50	1.45	1.49	1.43	1.29	1.10	1.18	0.99	1.22	0.71

*N* = 412, **p < 0.01, ***p < 0.001.

### 3.3. Hypotheses testing

**Figure 2:**
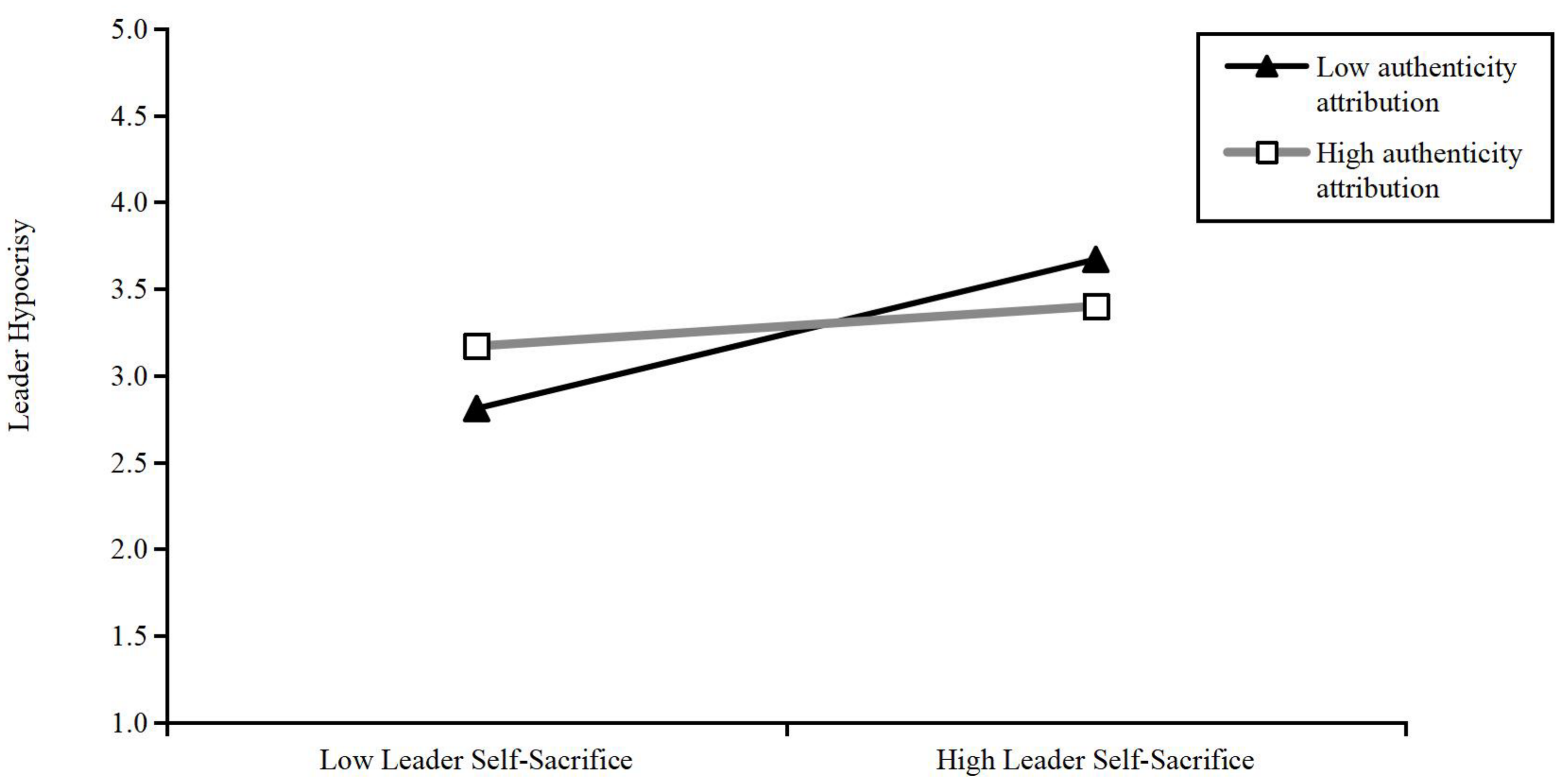
Moderating effect of authentic attribution in the relationship between leader self-sacrifice and leader hypocrisy.

**Figure 3:**
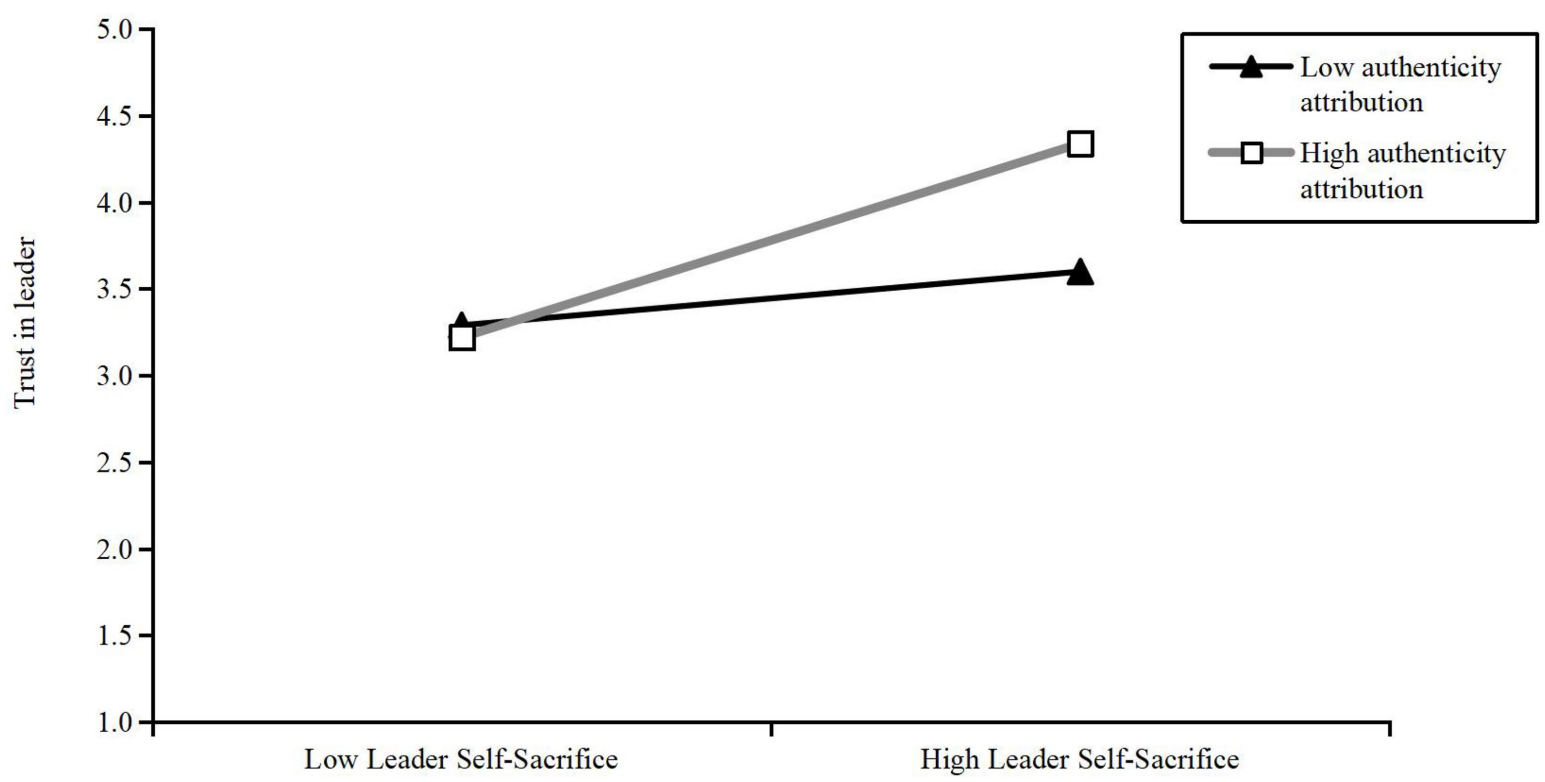
The moderating effect of authenticity attribution in the relationship between leader self-sacrifice and leader trust.

We used Model 7 of the SPSS macro program compiled by Hayes to test the interaction effect, with a random repeated sample set to 5,000. As shown in Table 3, we first tested the interaction effect between leader self-sacrifice, employee authenticity attribution, and leader hypocrisy. The product of employee authenticity attribution and leader self-sacrifice had a significant negative impact on leader hypocrisy (*b* = −0.12, *p* < 0.01), with a 95% confidence interval of [−0.21, −0.04], excluding 0. As illustrated in Figure 2, the simple slope analysis showed the following: When employee authenticity attribution was high, the interaction between leader self-sacrifice and employee authenticity attribution had no significant effect on leader hypocrisy (*b* = 0.09, t = 1.33, ns), with a 95% confidence interval of [−0.04, 0.21], including 0; when employee authenticity attribution was low, the interaction between leader self-sacrifice and authenticity attribution positively affected leader hypocrisy (*b* = 0.33, *t* = 5.43, *p* < 0.001), with a 95% confidence interval of [0.21, 0.45], excluding 0. This verified H1. Second, we tested the interaction effect between leader self-sacrifice, employee authenticity attribution, and leader trust. The product of authenticity attribution and leader self-sacrifice had a significant positive effect on leader trust (*b* = 0.16, *t* = 4.06, *p* < 0.001), with a 95% confidence interval of [0.08, 0.23], excluding 0. As illustrated in Figure 3, the simple slope analysis showed the following: When employee authentic attribution was high, the interaction between leader self-sacrifice and authentic attribution positively affected leader identification (*b* = 0.43, *t* = 2.18, *p* < 0.001), with a 95% confidence interval of [0.32, 0.54], excluding 0; when employee authenticity attribution was low, the interaction between leader self-sacrifice and employee authenticity attribution had a weak effect on leader trust (*b* = 0.12, *t* = 2.18, *p* < 0.05), with a 95% confidence interval of [0.01, 0.23], excluding 0. This verified H3.

**Table 3:** Interaction effect test.

Variables	Leadership hypocrisy			Trust in leader		
	*b*	*SE*	*t*	*b*	*SE*	*t*
Constant	3.13	0.21	14.98	3.70	0.19	19.85
Control variables						
Sex	−0.01	0.11	−0.06	0.04	0.10	0.45
Age	0.03	0.04	0.79	−0.01	0.03	−0.14
Education	−0.01	0.04	−0.17	−0.05	0.03	−1.55
Working time with supervisor	0.02	0.04	0.55	0.02	0.03	0.56
Independent variable						
Leader self-sacrifice	0.21^***^	0.05	4.57	0.27^***^	0.04	6.76
Authenticity attribution	0.02	0.06	0.40	0.17^**^	0.06	3.01
Interaction						
Leader self-sacrifice× Authenticity attribution	−0.12^**^	0.04	−2.84	0.16^***^	0.04	4.06
*R^2^*	0.09			0.16		
*F*	5.71^***^			11.31^***^		

**p < 0.01, ***p < 0.001.

We used the SPSS Process macro program to set a random repeated sample of 5,000 for the regression analysis. The results showed that leader hypocrisy negatively affected employee organizational citizenship behavior (*b* = −0.14, *t* = −4.66, *p* < 0.001), with a 95% confidence interval of [−0.20, −0.08], excluding 0. Meanwhile, leader trust positively affected employee task performance (*b* = 0.31, *t* = 5.47, *p* < 0.001), with a 95% confidence interval of [0.20, 0.42], excluding 0. This preliminarily validated H2 and H4. To further validate H2 and H4, we used the Bootstrap indirect effect test as a conditional process analysis (Hayes and Rockwood, 2020). Table 4 shows the results. With high employee authenticity attribution, the indirect effect of leader self-sacrifice and employee organizational citizenship behavior through leader hypocrisy was not significant. The mediating effect value was−0.01, with a 95% confidence interval of [−0.03, 0.01], including 0. With low employee authenticity attribution, the indirect effect of leader self-sacrifice and employee organizational citizenship behavior through leader hypocrisy was significant. The mediating effect value was −0.05, with a 95% confidence interval of [−0.07, −0.02], excluding 0. The difference between groups was 0.03, with a 95% confidence interval of [0.01, 0.06], excluding 0. Further, the calculated index was 0.02, not 0, with a 95% confidence interval of [0.01, 0.03]. This fully verified H2. With high employee authenticity attribution, the indirect effect of leader self-sacrifice and employee task performance through leader trust was significant. The mediating effect value was 0.13, with a 95% confidence interval of [0.08, 0.20], excluding 0. With low employee authenticity attribution, the indirect effect of leader self-sacrifice and employee task performance through leader trust was significant. The mediating effect value was 0.04, with a 95% confidence interval of [−0.01, 0.08], excluding 0. The difference between groups was 0.10, with a 95% confidence interval of [0.04, 0.17], excluding 0. Finally, the calculated index was 0.05, with a 95% confidence interval of [0.02, 0.08], excluding 0. This fully verified H4.

**Table 4:** Conditional process analysis test results.

Path	Authenticity attribution	b	BootSE	Bootstrap (95%CI)
Leader self-sacrifice → leadership hypocrisy → organizational citizenship behavior	High	−0.01	0.01	[−0.03, 0.01]
	Low	−0.05	0.01	[−0.07, −0.02]
Differences in values	High minus low	0.03	0.01	[0.01, 0.06]
Leader self-sacrifice → Trust in leader→ Task performance	High	0.13	0.03	[0.08, 0.20]
	Low	0.04	0.02	[−0.01,0.08]
Differences in values	High minus low	0.10	0.03	[0.04, 0.17]

## 4. Discussion

Based on attribution theory, this study explored the double-edged sword effect of leadership self-sacrifice behavior on employees’ work results *via* two paths from the perspective of employees’ attributions of the authenticity of this behavior. Concretely, our study explored not only the negative impact of leadership self-sacrifice behavior, but also takes into account its proven positive impact. There were four main findings. First, employees perceived leader hypocrisy under low authenticity attributions of leader self-sacrifice behavior. Second, employees developed trust in leaders under high authenticity attributions of leadership self-sacrifice behavior. Third, leader hypocrisy had a negative mediating effect in the relationship between employee authenticity attribution, leadership self-sacrifice, and employee organizational citizenship behavior; here, the mediating effect was stronger when employees reported lower authenticity attributions. Fourth, trust in leaders had a positive mediating effect in the relationship between employee authenticity attribution, leadership self-sacrifice, and employee task performance; here, the mediating effect was stronger when employees reported higher authenticity attributions. Based on this evidence, we outline several theoretical and practical implications below.

### 4.1. Theoretical implications

It is the first time to explore the positive and negative impacts of leaders’ self-sacrifice behavior on employees’ work outcomes to promote and enrich the research on this type of behavior. In the traditional cognition field and previous related research, scholars have generally posited that leadership self-sacrifice behavior can effectively motivate and inspire employees, thereby inducing good work results (Yang F. et al., 2022). However, this view seems to be one-sided as leadership self-sacrifice behavior not only involves leaders themselves but is also subjected to employees’ attributions and interpretations (Dasborough and Ashkanasy, 2002). Taking the perspective of followers through attribution theory, this study investigated the negative side of leadership self-sacrifice behavior, with a focus on employees’ attributions of its overall authenticity, thus revealing a double-edged sword effect. Moreover, this study also constitutes a response to Martinko et al. (2011), who called for more research involving the subordinate perspective on attribution in the organizational context.

Second, we found important boundary conditions to the influence of leadership self-sacrifice behavior on employees. In this regard, our findings emphasize that employee attributions play a critical role in the process by which leaders influence their subordinates. This also adds new evidence to the literature as scholars have only recently begun to explore how leadership behavior impacts employees from the perspective of employee attribution (Qin et al., 2020; Xing et al., 2021). For example, some scholars have explored how leader humility impacts employee work outcomes from the perspective of employee impression management attribution (Bharanitharan et al., 2021). Although Bolino and Turnley (1999) reported that individual self-sacrifice behavior constitutes an impression management strategy when dividing the individual impression management dimension, this may easily arouse suspicions about behavioral authenticity. Choi and Mai-Dalton (1998) similarly suggested that leaders’ self-sacrifice behavior may be inauthentic. However, we know of no previous studies that have investigated leader self-sacrifice behavior from the perspective of employee authenticity attribution. We propose that the impact of leadership self-sacrifice behavior on employees depends on employees’ attributions of authenticity to this leadership behavior. It is precisely due to different attribution results that employees experience the two different psychological states of trusting leadership and perceiving leadership hypocrisy, which, in turn, affects their subsequent work results. This means that the drivers behind the leadership of self-sacrifice behavior need to be considered.

Third, under different levels of authenticity attribution, we found that perceptions of leader hypocrisy and trust in leaders mediated the relationship between leader self-sacrifice behavior and employee work results. While scholars have made some progress pertaining to the dark side of leadership, the research on leader hypocrisy has not yet aroused widespread concern or discussion. In particular, no studies have explored the relationship between leader self-sacrifice and leader hypocrisy. From the perspective of subordinates’ authenticity attributions of leadership behavior, this study revealed the mediating role of leader hypocrisy, thus enriching the literature on that factor. Based on social exchange theory, some scholars have explored how trust in leadership mediates the relationship between leader self-sacrifice and employee work outcomes (Chen et al., 2020), but they have not tested employees’ perceptions and attributions of leadership behavior. We addressed this omission by explaining and clarifying the mediating role of trust in leaders from different perspectives.

### 4.2. Practical implications

From the perspective of employee attribution, this study explored the potential negative impact of leaders’ self-sacrificial behavior and its double-edged sword effect. The study’s findings have practical significance for managers who aim to understand leaders’ self-sacrificial behavior. First, this study holds that although displays of leaders’ self-sacrifice can be used to motivate and inspire employees, it should not be abused. Since it is an uncommon behavior, frequent use of self-sacrifice can place psychological and work pressure on employees (Choi and Mai-Dalton, 1998), may take on the form of the moral coercion of employees, and may induce employees to doubt leaders’ authenticity. Therefore, we suggest that leaders take the lead but that they do not demonstrate self-sacrifice frequently, only when facing difficulties or at critical moments. Second, it must be considered that since leaders sometimes deliberately show self-sacrifice in order to manipulate employees, employees could also perform impression management through the display of self-sacrifice behavior (Bonner et al., 2017). We believe that this possibility exists, even in management practice. Therefore, we propose that leaders should privately praise employees who show self-sacrifice behavior, as appropriate, but should not encourage or publicize them because self-sacrifice implies a denial of self-subjectivity, which means that collective interests need to be above self-interest. Vigorously promoting and encouraging this behavior can put additional pressure on other employees, which is unfair to them.

### 4.3. Study limitations and directions for future research

This study has some limitations. First, although it used multi-source and multi-time data collection methods, our variables were measured through self-administered questionnaires, meaning it was impossible to completely avoid potential common method bias. Future studies can employ situational experiments to explore the relationship between leader self-sacrifice behavior and employee work outcomes. Second, due to resource constraints, the study samples were limited to mid-level leaders and their subordinates. However, senior leaders within teams or organizations are generally more likely to show self-sacrifice behavior. Thus, future studies can use trickle-down theory to explore the work results of mid-level leaders and their subordinates when senior leaders show self-sacrifice behavior. Third, compared with Western leaders, Eastern leaders may advocate greater self-sacrifice. In this regard, our effect value may be relatively higher. In the future, we hope to verify whether our conclusions can also be established in the Western context. Fourth, the research variables selected in this study were at the individual level and, thus, could not reveal the impact of organizational context or organizational atmosphere on the employee attribution process and its results. Future research should incorporate organizational variables into the research framework. Finally, the current operating conditions of an organization may affect employees’ perceptions of leaders’ self-sacrificial behavior; however, we failed to incorporate this aspect into the present study. This should be considered in future studies.

## Data availability statement

The raw data supporting the conclusions of this article will be made available by the authors, without undue reservation.

## Ethics statement

Ethical review and approval was not required for the study on human participants in accordance with the local legislation and institutional requirements. Written informed consent for participation was not required for this study in accordance with the national legislation and the institutional requirements.

## Author contributions

Y-CJ and Y-CW drafted and designed the work. Y-CJ collected the data and drafted the manuscript. Y-CW analyzed the data. They all revised the manuscript. All authors gave final approval of the manuscript before submission.

## Funding

This work was supported by the Gansu Excellent Graduate Student Innovation Star Project of China (Grant No. 2022CXZX-725), Gansu Provincial Department of Science and Technology Soft Science Fund Project of China (Grant No. 21CX6ZA101), and General Projects of Lanzhou University of Finance and Economics of China (Grant No. Lzufe2021C-016).

## Conflict of interest

The authors declare that the research was conducted in the absence of any commercial or financial relationships that could be construed as a potential conflict of interest.

## Publisher’s note

All claims expressed in this article are solely those of the authors and do not necessarily represent those of their affiliated organizations, or those of the publisher, the editors and the reviewers. Any product that may be evaluated in this article, or claim that may be made by its manufacturer, is not guaranteed or endorsed by the publisher.
